# Selective Gene Expression by Postnatal Electroporation during Olfactory Interneuron Neurogenesis

**DOI:** 10.1371/journal.pone.0001517

**Published:** 2008-01-30

**Authors:** Alexander T. Chesler, Claire E. Le Pichon, Jessica H. Brann, Ricardo C. Araneda, Dong-Jing Zou, Stuart Firestein

**Affiliations:** 1 Department of Biological Sciences, Columbia University, New York, New York, United States of America; 2 Department of Biology, University of Maryland, College Park, Maryland, United States of America; University of Washington, United States of America

## Abstract

Neurogenesis persists in the olfactory system throughout life. The mechanisms of how new neurons are generated, how they integrate into circuits, and their role in coding remain mysteries. Here we report a technique that will greatly facilitate research into these questions. We found that electroporation can be used to robustly and selectively label progenitors in the Subventicular Zone. The approach was performed postnatally, without surgery, and with near 100% success rates. Labeling was found in all classes of interneurons in the olfactory bulb, persisted to adulthood and had no adverse effects. The broad utility of electroporation was demonstrated by encoding a calcium sensor and markers of intracellular organelles. The approach was found to be effective in wildtype and transgenic mice as well as rats. Given its versatility, robustness, and both time and cost effectiveness, this method offers a powerful new way to use genetic manipulation to understand adult neurogenesis.

## Introduction

In the central nervous system, postnatal neurogenesis persists into adulthood and supplies a restricted subset of interneurons in two tissues: the olfactory bulb and the hippocampus [1]. The most robust of these appears to be in the olfactory bulb, where inhibitory interneurons are generated at the astounding rate of 30-50,000 per day in the mouse [2]. These cells are born from a population of progenitor cells located in the Subventricular Zone (SVZ) and then migrate to the bulb along a path known as the rostral migratory stream (RMS). Within the olfactory bulb, migrating neuroblasts give rise to a diversity of interneurons that mediate local signal processing. Among them are periglomerular cells, a class of interneurons that modulate the transmission of sensory stimuli at the first synapse in olfactory processing, and granule cells which modulate the output through numerous synapses with the projection neurons, mitral cells.

Many investigators have been drawn to study postnatal neurogenesis in the olfactory bulb and the hippocampus to address a variety of questions surrounding this important and unusual process. What determines the fate of precursor cells in the SVZ and RMS? How do the postnatally generated cells integrate into existing circuits? What determines whether they will survive? What controls rates of proliferation? How is this balanced with rates of cell death? Are there age related factors that are important? Investigating these and other fundamental questions require the ability to track cells from birth to maturity, to monitor activity, and to alter gene expression in individual cells.

Traditionally, transgenic or gene targeted mice have proved a powerful strategy for the manipulation of gene expression, but suffer from being time consuming and expensive. Viruses have also proven useful, but are difficult to generate in high titers, have limitations in terms of insert and promoter size, and have associated biohazards that require special handling. More recently, electroporation of genes in rodents has been gaining as an alternative to these techniques [3]–[5]. Electroporation has several advantages over other approaches: the plasmid construction is simple, versatile, rapid, and inexpensive, and it is applicable to numerous tissues and species [6]. Given the wealth of candidate genes generated from genomics and proteomics, ectopic expression or knockdown of genes by electroporation represents a powerful way to elucidate the roles of the numerous candidate molecules *in vivo*.

Thus far the use of electroporation in the brain has been largely limited to embryonic tissue. Injection of plasmids is typically performed *in utero*, where the delivery of the electrical pulse enables the constructs to enter cells [3]. However, this is a surgical technique with significant risks of pup mortality [5]. More recently, electroporation has been applied to the postnatal retina and cerebellum [4], [7]. In our efforts to understand postnatal neurogenesis we developed a highly efficient and non-invasive procedure that utilizes electroporation for manipulating gene expression in the subventricular zone (SVZ). Here we introduce a method of electroporation in early postnatal animals that requires neither surgery nor stereotaxic apparatus, but which results in widespread expression of foreign genes in specific cell populations, with nearly 100% success rates. This methodology makes genetic manipulation in rodents a bench top procedure, bringing it within the technical and financial reach of most laboratories.

## Results

**Figure 1:**
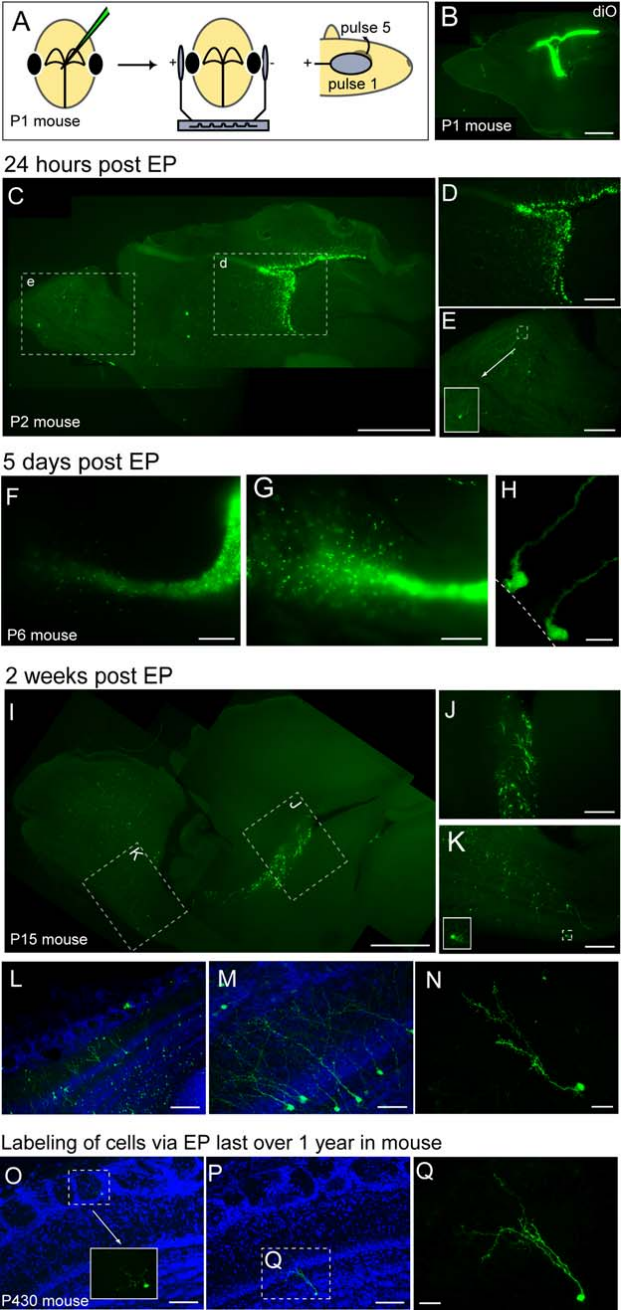
Long-term ectopic expression of GFP in neurogenic regions of the postnatal brain by electroporation. A. Animals immobilized by cooling are injected with plasmid using a dissecting microscope and picospritzer. The injection is made rostral to the olfactory bulb followed by a series of 5 voltage pulses delivered with tweezer-type electrodes. The pulses are given while sweeping the positive pole upwards from the eyes by 45 degrees. B. Sagittal view of a P1 mouse showing localized fluorescence after injection of diO into the lateral ventricles. Scale = 1 mm. C–E. Sagittal views of a P2 mouse 24 hours post-electroporation of the olfactory bulb and ventricle in a P2 mouse 24 hours post-electroporation. C. The preponderance of GFP is restricted to the ventricular wall; 60 µm sections, scale = 1 mm. D–E. Enlarged views of the boxed regions in c highlight the high levels of expression in along the SVZ relative to the olfactory bulb. Relatively few GFP positive cells can be found in the olfactory bulb (for example see inset in E). scale = 500 µm. F–G. Sagittal views of a P6 mouse 5 days post-electroporation. F. Whole-mount view of an unfixed brain reveals high levels of GFP expression through the RMS. Scale = 1 mm G. A higher magnification view of a different brain showing cells had begun to migrate throughout the olfactory bulb. Scale = 500 µm H. Labeled radial glia persisted in the SVZ; dashed line denotes ventricular wall; 60 µm sections, scale = 50 µm. I–N. Sagittal views of a P15 mouse 2 weeks post-electroporation; 60 µm sections I. In addition to the RMS, GFP-positive cells can be found throughout the olfactory bulb. scale = 1 mm. J–K. Enlarged views of the boxed regions in i showing the RMS (J), and olfactory bulb (K). Inset in K highlights a periglomerular cell. scale = 500 µm. L–N. Z-projections of 60 µm sections TOTO3 staining reveals nuclei (blue). L. Widespread distribution of GFP positive cells in all layers of the bulb. Scale = 200 µm M. Labeling of numerous granule cells. scale = 50 µm N. Resolution of a single morphologically mature granule cell. scale = 50 µm. O–Q. Sagittal views of a P430 mouse over 1 year post electroporation; 60 µm sections. O. labeled PG cell, TOTO 3 (blue) stains all nuclei and inset show a magnified view. Scale = 100 µm P–Q.Labeled granule cell, low magnification counterstained with TOTO3 (P; scale = 100 µm) and high magnification of the same cell (Q; scale = 25 µm)

We investigated the use of *in vivo* electroporation as an alternative to viral and transgenic methods for the study of postnatal neurogenesis. With the aid of a dissecting microscope we found the illumination of a fiber optic light sufficient for the visualization of the olfactory bulbs and central sulcus of the brain. Under such conditions we reproducibly injected lateral ventricles of mice ages P0-P4 without the requirement of surgery or a stereotactic device (figure 1a and 1c). A sharp electrode with a 20–50 µm beveled tip was used to pierce through the skin and developing skull into the brain and inject 1–2 µL of endotoxin-free plasmid (1–5 µg/µL) into the ventricles. Similar to published methods both *in utero* and in postnatal retinas, we determined that a 5 second square pulse protocol of five 50 ms pulses of 150 V gave optimal results with minimal side effects [4]. The first pulse was aligned with the center of the eyes and subsequent pulses were given while sweeping the positive pole 45 degrees upwards (figure 1a). Electroporation of a plasmid encoding EGFP under the CAG promoter resulted in high levels of fluorescence within 24 hours (figure 1c–d). The preponderance of fluorescence from animals injected was found in the SVZ compared with the OB (compare figure 1d and 1e).

The pulse protocol resulted in robust ectopic expression with no detectable adverse effects on pup survival. A litter of 8 pups could be anesthetized on ice, injected, and returned to their mother in less than 10 minutes with nearly all (>95%) surviving to adulthood and expressing the plasmid of interest. The robustness and ease of our approach offers a major improvement over *in utero* electroporation in that the latter requires surgery on expensive timed-pregnant animals that can lead to significant damage and loss of embryos. Our approach was also remarkably specific, exclusively labeling the postnatal neurogenic region of the mouse brain.

Neuroprogenitors in the SVZ give rise to neuroblasts that migrate along the rostral migratory stream (RMS). 5 days post-electroporation we found strong GFP expression in the RMS (figure 1f). Cells that had reached the bulb radially migrated throughout the bulb (figure 1g). GFP positive cells lining the ventricles were also noted, indicating the persistence of labeled progenitor cells (figure 1h). Migrating neuroblasts terminally divide to generate the interneurons in the olfactory bulb. 2 weeks post-electroporation we continued to observe labeling in the SVZ and in the RMS (figure 1i–j) but now also found interneurons in all layers of the olfactory bulb (figures 1i and 1k). Postnatal electroporation rivaled viral techniques, in that it was sufficient for widespread labeling of both granule and periglomerular cells (figure 1l–m) as well as for resolution of single cells (figure 1n). Numerous GFP-positive cells could be found several months post-electroporation and, although less numerous, persisted in mice after over a year (figure 1o–q).

**Figure 2:**
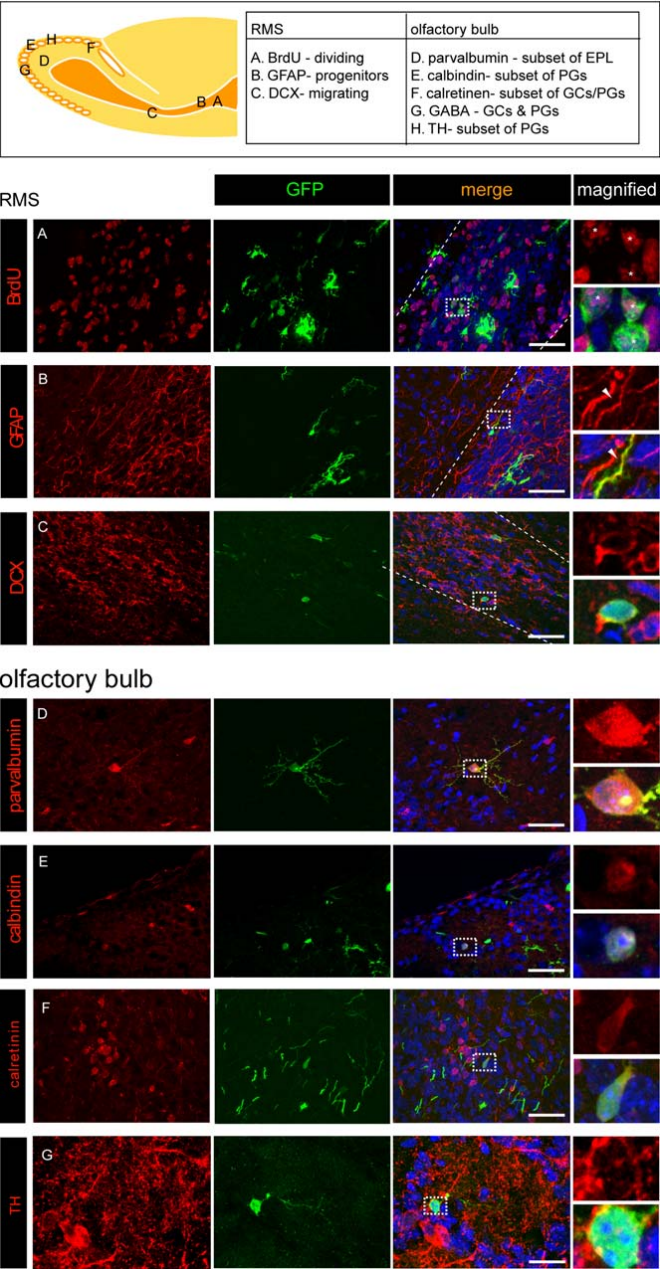
Widespread labeling of specific cell populations by electroporation. 14 µm sections from a P15 mouse electroporated 2 weeks previously with GFP (green) were stained with a panel of markers (red) labeling several specific cell populations in RMS (A–C) and olfactory bulb (D–G). 10-fold magnified views are presented for highlighted double-labeled cells. A summary of the locations for each section and the markers used are in the box on top. scale = 50 = µm

We found labeling in every specific cell population we analyzed. A subset of GFP-positive neuroprogenitors of the SVZ and RMS were co-labeled by a brief intraperitoneal pulse of BrdU (figure 2a). Furthermore, we observed GFP expression among the GFAP-positive progenitors (figure 2b) and among the doublecortin-positive migrating neuroblasts in the RMS (figure 2c). SVZ progenitors are a heterogeneous population that gives rise to a diversity of interneurons in the olfactory bulb [8]–[9]. Labeled neurons in the bulb included both granule and periglomerular cells. We found overlap between GFP and each cell-type specific marker analyzed (parvalbumin, calbindin, calretinin, and tyrosine hydroxlyase). Notably, co-staining for all markers tested could be found from sections prepared from a single animal (figure 2d–g) showing that electroporation labels diverse progenitor populations within the SVZ.

**Figure 3:**
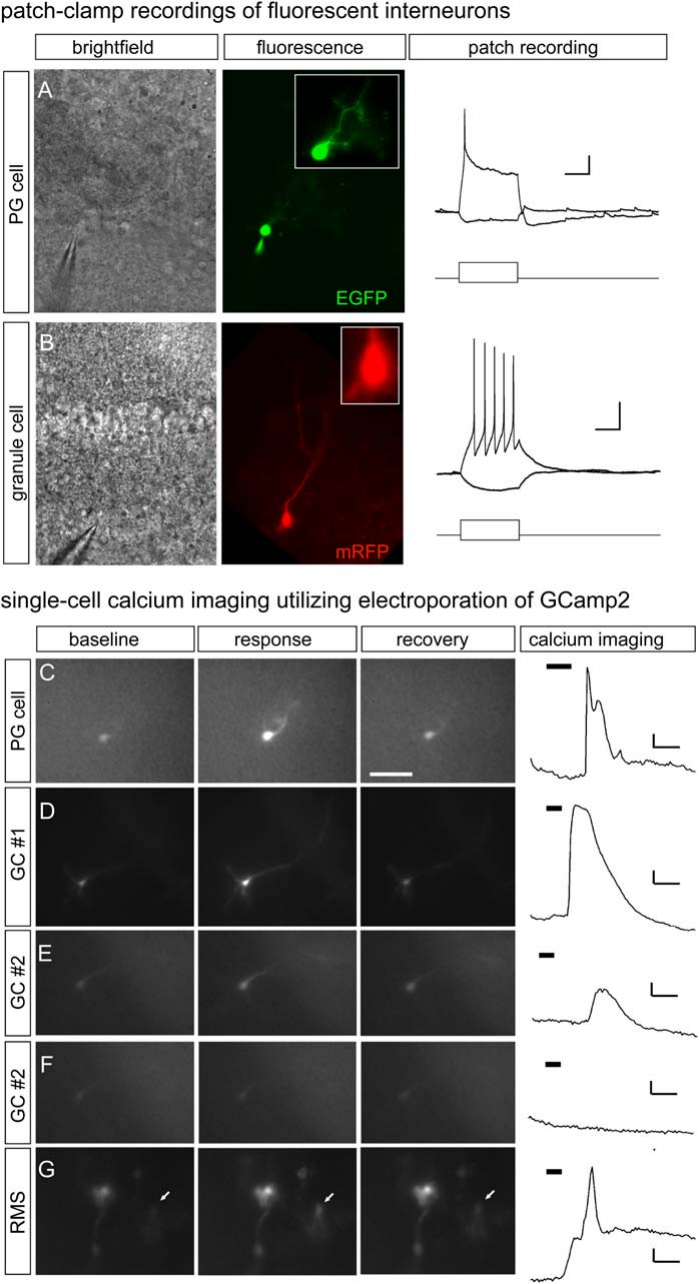
Electrical and optical recordings from interneurons labeled by electroporation. Electrical excitability of neurons in current-clamp recordings in slices from 3–4 weeks-old mice electroporated with EGFP (A) and mRFP (B). A. Example of PG cell recorded in the glomerular layer (bright field, left) exhibited spontaneous synaptic activity and responded to a depolarizing current (16 pA) with a single action potential followed by an afterhyperpolarization. The cell input resistance is 1 GΩ as measured with a negative current stimulus (−4 pA). B. Example of a granule cell recorded in the internal plexiform layer (bright field, left). This cell responded to a depolarizing stimulus (15 pA) with a train of spikes. The cell input resistance is 1.8 GΩ as measured with a negative current stimulus (−5 pA). The calibration in A and B is 200 ms; 10 and 20 mV respectively. C–G. Calcium imaging of single cells in slice from 3–6 week old mice electroporated with pCAG-GCamp2. C. PG cell before, during, and after response to KCl (100 mM) presented during frames 10–25 (36–96 seconds; total of 1 min). Scale bar, 100 mm. (frames 5, 35, and 60 or 16, 136, 236 sec). Scale bar, 5%ΔF/F, 1 min. D. Granule cell (at the border of the IPL/Mitral cell layer) responds to KCl (100 mM) presented during frames 10–20 (36–76 seconds; total of 40 sec). Scale bar = 3%ΔF/F, 1 min. E. A different granule cell (at the border of the IPL/Mitral cell layer) responds to glutamate (100 µM) presented during frames 10–20 (36–76 seconds; total of 40 sec). Scale bar = 2%ΔF/F, 1 min. F. Granule cell (same as in e) does not respond to Ringer presented during frames 10–20 (36–76 seconds; total of 40 sec). Scale bar = 2% ΔF/F, 1 min. G. Migrating cell in RMS during and after response to KCl (100 mM) presented during frames 10–20 (36–76 seconds; total of 40 sec). Cell showed no response to application of Ringer alone (not shown). Scale bar = 5%ΔF/F, 1 min

Cells ectopically expressing GFP divide, migrate, and morphologically mature. The ease, rapidity, cost effectiveness, and robustness of the technique suggested postnatal electroporation to be an ideal approach to study many aspects of neurogenesis, cell migration, circuit integration, and neurophysiology. Importantly, in addition to appearing morphologically healthy, we found that fewer than 0.5% of GFP+ cells were also labeled by activated caspase 3 antibodies or TUNEL, showing that electroporation does not accelerate cell mortality. Additionally we found neurons labeled via electroporation also had the expected electrical properties. We characterized the membrane properties of periglomerular (figure 3a) and granule cells (figure 3b) expressing fluorescent markers (mRFP or GFP) by performing patch clamp recording in acute bulb slices. Labeled cells were electrophysiologically indistinguishable from neurons derived from non-electroporated animals. Mature granule and periglomerular cells were recognized by their morphology and their position in different layers of the olfactory bulb. In current-clamp recordings granule cells had a resting membrane potential of 65±2 mV and input resistance of 1.57±0.22 GΩ (n = 6). Depolarizing stimuli (2–10 pA) elicited action potentials, which increased in frequency with larger currents. Mature periglomerular cells cells, like granule cells had high input resistance (1.12±0.58 GΩ) and hyperpolarized membrane potentials (67±2 mV), but unlike granule cells, increasing depolarizing stimuli produced a single spike followed by a plateau potential (n = 3). In agreement with these observations, in voltage-clamp both periglomerular cells and granule cells exhibited both voltage-dependent inward and outward currents (not shown). In addition, we recorded from a subpopulation of cells at various stages of development (as assessed by their dendritic morphology); most of these cells exhibited small voltage-dependent inward currents and depolarizing stimuli failed to induce all-or none action potentials in current-clamp (not shown). Similar characteristics for granule and periglomerular cells have been described both in control [10], as well as in retrovirally GFP-labeled cells [11]-[12]. Our results also demonstrate the feasibility of using recording from postnatally born neurons genetically manipulated by electroporation to study their functional properties.

In addition to monitoring cells electrically, we also developed tools to monitor activity optically. Genetically encoded sensors offer a non-invasive tool to study neuronal function *in vivo*, are less technically demanding and time consuming than the use of single-cell electrophysiology, and allow unequivocal identification of morphology. Furthermore, unlike transgenic approaches, electroporation of a sensor would be ideal to resolve the detailed morphology of single cells both in slices and *in vivo*. Toward this end, we created a plasmid (pCAG-GCamp2) allowing expression of the GFP based fluorescent calcium reporter [13]. GCamp2 has been reported to be a sensitive reporter of calcium transients on par with dye-based sensors, and its transgenic expression in mice has been shown to lack any discernable deleterious effects [13]–[16].

Examination of mice electroporated with pCAG-GCamp2 revealed a distribution of fluorescent cells similar to GFP controls. GCamp2-expressing cells migrated to destinations throughout the olfactory bulb where they were found to be morphologically mature. All cell types labeled were able to respond with an increase in fluorescence to high potassium (Figure 3c, d) as well as the physiological ligand, Glutamate (Figure 3e, g), but did not respond to Ringer application (Figure 3f; N = 19 slices from 4 animals). In addition, we were able to monitor intrinsic calcium waves found in immature cells in the RMS of the OB (see Supplemental Movie S1). Calcium imaging was also highly reliable; cells were responsive to repeated applications of stimuli (data not shown) and to a variety of stimuli.

**Figure 4:**
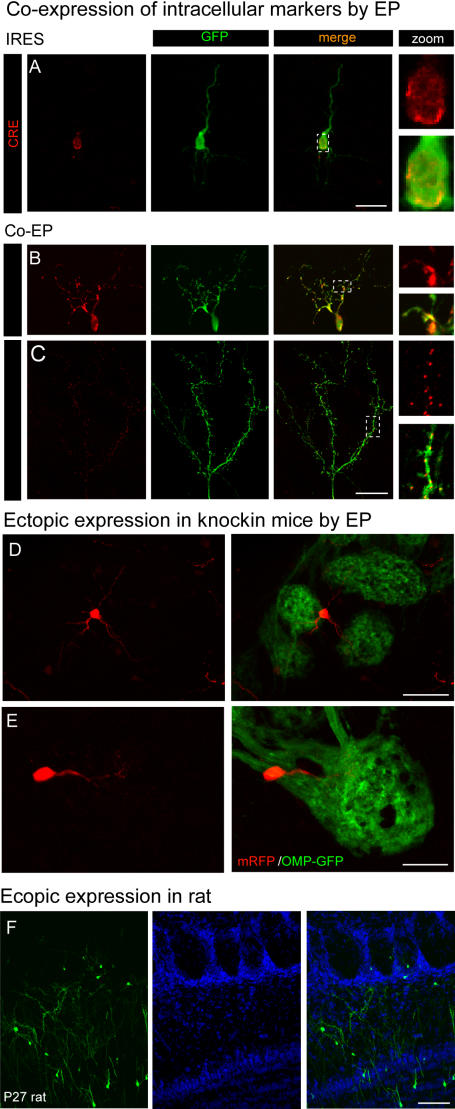
Electroporation is effective for the expression of multiple constructs, in transgenic animals, and in rats. A. 20 µm section of an olfactory bulb of a P6 mouse 5 days post-electroporation with pCAG-CRE-ires-EGFP; anti-CRE staining labels the nucleus (red) and GFP (green) labels the cytoplasm with a 10X magnified view of the cell body. Scale = 25 µm. B–C. Z projections of 60 µm sections from olfactory bulbs co-electroporated with GFP and either dsRED-Golgi (B; 5 days post-electroporation) or dsRED-ER (C; 14 days post electroporation) showing labeled organelles. 10X magnified views of highlighted regions show perinuclear golgi staining in the cell body and punctate ER staining at the base of dendritic spines. Scale = 25 µm. D–E. Electroporation of OMP-GFP mice with RFP plasmid. Z-projections of 60 µm sections showing the morphology of single periglomerular cells (red) and the glomeruli they innervate (green). 2 types of PG cells are shown, one that innervates 3 glomeruli (E; scale = 25 µm) and another other with its processes are confined to a single glomerulus (F; scale = 50 µm). F. Highly efficient electroporation of rats. Z-projection of 60 µm section from a P27 rat olfactory bulb electroporated at P1 showing widespread ectopic expression in all layers of the olfactory bulb. TOTO3 (blue) labels all nuclei. Scale = 50 µm.

We also used electroporation to simultaneously express multiple genes. We demonstrated co-expression by coincident marking of various intracellular compartments (figure 4a–4c). First we co-expressed CRE and GFP using a single plasmid containing an internal ribosome entry site (pCAG-CRE-IRES-GFP). Antibody staining of electroporated animals produced GFP-positive cells with CRE labeling in their nuclei (figure 4a). Alternatively, we found co-electroporation to be equally effective. Co-electroporation of two plasmids, one encoding GFP and the other either dsRed-Golgi or dsRed-ER allowed visualization of these organelles without using antibodies both *in vivo* (data not shown) and in fixed tissue (figures 4b–c). We found near perfect overlap when a 1∶1 ratio of plasmids was used. While GFP expression labeled the entirety of the cell, both dsRed-Golgi and dsRed-ER localized to perinuclear rings in the cell body (figure 4b and data not shown). Expression of dsRed-Golgi also labeled the dendrites with highly concentrated signal at branch points (figure 4b inset). Punctate dsRED-ER fluorescence was also observed throughout the dendritic arbors of interneurons (figure 4c). Consistent with reported roles for local protein synthesis in spine morphogenesis and synapse formation, the puncta of dsRed-ER fluorescence localized to the base of dendritic spines (figure 4c inset). Given the importance of protein synthesis and trafficking in dynamic neural circuits, *in vivo* imaging of these organelles should offer insight into the regulation of these processes [17]–[18].

One significant advantage over *in utero* electroporation is that performing the procedure postnatally obviates the requirement of surgery on timed pregnant mice. *In utero* electroporation is challenging in transgenic or knockin mouse strains that have smaller litters, are more difficult to breed in large numbers, and are more sensitive to surgical manipulations. These problems are overcome by performing electroporation postnatally where it was as efficient in knockin mice as wild type strains (figure 4d–e). We took advantage of an existing strain with fluorescently labeled olfactory sensory neuron axons, synaptic partners of periglomerular cells [19]. OMP-GFP mice electroporated with mRFP allowed us to resolve the unique morphology of periglomerular cell subtypes including their arbors within GFP-positive glomeruli (figure 4d–e). Finally, beyond its utility in various mouse strains, electroporation is also efficient in other mammals. Similar to other studies, we found the technique was equally effective in rats, thus broadening its application to species lacking good genetic tools (figure 4f).

## Discussion

Numerous methods to alter gene expression in cells of living animals have been developed in the last decade or so and many are now in current use. Some still require significant technical sophistication, while others are more amenable to common lab practices. In line with these latter procedures we have here described a technique that produces reliable results with a minimum of technical sophistication in molecular biology or genomics. This should make the ability to alter gene expression available to laboratories normally focused on physiology or developmental studies.

Among the critical attributes of a gene alteration technique are reliability and specificity. Control of gene expression is most useful when it can be specified in particular cells or cell types. With transgenesis this is usually accomplished by using a cell specific promoter, if one is available. In the case of electroporation some specificity can be gained by targeting the plasmids to certain brain regions, by “aiming” the electrical pulse, or by using the procedure at specific developmental time points. Here we have made use of all three of these strategies, resulting in gene expression in a limited set of interneuronal cells in the olfactory bulb.

In particular our interest here has been to follow the population of proliferating cells in the SVZ and the integration of their daughter neurons into existing circuits in the olfactory bulb. Not only does this technique allow us to follow individual cells over extended periods of time, but we have also used functional markers that permit physiological recordings in brain slices. We demonstrate that GCamp2 is functionally expressed in periglomerular and granule cells of the olfactory bulb, thus providing an opportunity to observe the response behavior of a large population of cells simultaneously. The role of these cells in sensory processing has remained controversial for some time and it might be expected that observing population responses will advance that understanding. Finally the expression of fluorescent markers results in the cell's entire morphology being visible – even in living tissue. Although beyond the scope of this communication, it should be possible to alter phenotypic characters and function by introducing new genes or by knockdown of genes with shRNA through electroporation. Additionally, we have demonstrated that the procedure can be performed in transgenic mice, allowing for the ability to knockout of floxed genes by electroporation of CRE, or the rescue of targeted disruptions in specific subpopulations. Further control over expression can be attained through the use of plasmids with temporal control elements and cell specific promoters [20].

The use of electroporation of the brain has become increasingly common, but has been generally limited to *in utero* delivery or labeling of single cells postnatally [21]. With the demonstration that electroporation can be used in early postnatal animals with high efficiency an important barrier to its widespread use has been removed. No surgery is required and there is no need to procure costly timed pregnant animals. Additionally, electroporation is applicable in a wide range of species. When a new technique can find widespread use further developments are likely to proceed at a rapid pace. We anticipate this will be the case with postnatal electroporation.

## Materials and Methods

### Postnatal electroporation

Newborn pups were anesthetized by hypothermia, placed under a dissecting scope and held by hand. Injection pipettes were beveled (300) on a grinding stone (Narashige EG-44) to have 20–50 µm openings Pipettes were guided into the brain rostral to the olfactory bulb using a micromanipular. 1–2 µL of endotoxin-free plasmid (1–5 µg/µL) were injected per pup. Immediately after injection pups were electroporated with tweezer type electrodes (BTX model 520) using a BTX ECM830. 5 pulses of 150V were given of 50 ms duration with a 950msec interval. Positive Electrode was swept 450 upwards from the first pulse aligned with the center of the eyes. After electroporation, pups were placed on a heat pad until they recovered full mobility and subsequently returned to the nest. SVE129 (Taconic) and OMP-GFP mice (kind gift of Peter Mombaerts, Rockefeller University) and Sprague Dawley rats (Taconic Farms) were housed according to Columbia University institutional animal care guidelines.

### Constructs

pCGLH (for expressing GFP) and pCRLH (for expressing mRFP) were kindly provided by Kenneth Kwan and Nenad Sestan (Yale University). To make pCAG-Gt, GFP was excised from pCGLH using EcoRI and replaced with the Gateway Reading Frame Cassette B (Invitrogen). GCamp2 was kindly provided by Junichi Nakai (RIKEN Brain Institute). A BglII-GCamp2-NotI fragment was subcloned into pENTR1a (Invitrogen) to create pENTR1a-GCamp2, and was subsequnetly recombined with pCAG-Gt to create pCAG-GCamp2. pCAG-Gt-IRES-EGFP was created by subcloning IRES-EGFPfor pIRES-EGFP2 (Clontech) behind the reading frame cassette. nlsCRE was kindly supplied by Andreas Walz (Rockefeller University), subcloned into pENTR1a and recombined into pCAG-Gt-IRES-EGFP. dsRED-Golgi and dsRED-ER (Clonetech) were subcloned into pENTR1a and recombined into pCAG-Gt.

### Tissue preparation and staining

Animals were anesthetized with ketamine/xylazine and transcardially perfused with 4% paraformaldehyde in PBS. Brain placed at 4°C, post-fixed for 4 hours and then incubated in 30% sucrose overnight. Tissue was mounted using TissueTek (Electron Microscopy Sciences, Hatfield, PA, USA) and section using a Leica CM1850 cryostat (Bannockburn, IL, USA). 60 µm sections were hydrated for 5 mins in PBS, incubated in TOTO-3 (1∶10,000; Molecular Probes) for 1 hour in 0.1% Triton-X in PBS (PBS-Tx), and washed 2 times in PBS. 14 µm sections were processed for immunohistochemistry by hydrating in PBS for 5 mins, blocking in 10% Normal Donkey Serum in PBS-Tx for 1 hour then incubating in primary antibody overnight (Mouse anti-BrdU (1∶50; Amersham), rabbit anti-GFAP (1∶1000; DAKO), goat anti-doublecortin (1∶500; Santa Cruz), mouse anti-parvalbumin (1∶1000, Sigma), mouse anti-calbindin (1∶1000; Sigma), mouse anti-calretinin (1∶1000, Chemicon), goat anti-tyrosine hydroxylase (1∶500; Santa Cruz)). After 4 washes in PBS-Tx, slides were incubated in secondary (1∶750 Alexa 488 and 594; Molecular Probes) for 2 hours. After washes, slides were mounted using Vectasheild, imaged on a confocal, and images analyzed using ImageJ.

### Slice preparation

Experiments were performed in olfactory bulb slices obtained from 3 to 4 week old mice. Animals were anesthetized with isofluorane and decapitated. Brain slices were prepared in a modified artificial cerebral spinal fluid (sucrose ACSF) of the following composition (in mM): 222 sucrose, 27 NaHCO_3_, 1.25 NaH_2_PO_4_, 3 KCl, 1 CaCl_2_ and 3 MgSO_3_. The whole brain was quickly removed and placed in oxygenated ice-cold sucrose ACSF. A block of tissue, containing part of the frontal lobes and the olfactory bulbs, was glued with cyanoacrylate to a microslicer stage and bathed in chilled sucrose ACSF. Sagittal sections (250–300 mm) of the olfactory bulb were sliced at using a vibrating microslicer (Leica VT1000). The slices were then transferred to an incubation chamber containing normal ACSF (see below) and left to recuperate first at 37°C for 30 min and then at room temperature for another hour. In all experiments, unless otherwise indicated, the extracellular solution is ACSF of the following composition (in mM): 125 NaCl, 25 NaHCO_3_, 1.25 NaH_2_PO_4_, 3 KCl, 2 CaCl_2_ and 1 MgSO_4_, 3 myo-inositol, 0.3 ascorbic acid, 2 Na-pyruvate and 15 glucose, continuously oxygenated (95% O_2_ and 5% CO_2_) to give a pH 7.4 and of osmolarity of ∼305 mOsm.

### Electrophysiological recordings

Slices were placed in a submerged recording chamber mounted on the stage of a Olympus BX51, upright microscope, fitted with infrared differential interference contrast optics (IR-DIC). Slices were observed with a 40X water immersion objective and labeled granule cells were recognized under fluorescent light (LG222 Sutter, CA). All experiments were performed at room temperature. Images of labeled cells were acquired using a CCD HQ2 camera (Photometrix). Standard patch pipettes (3–7 MW resistance) were pulled on a horizontal puller. Cells were recorded in the current-clamp mode the internal solution had the following composition (in mM); 135 K-gluconate, 10 NaCl, 10 KCl, 10 Hepes-Na, 2.5 ATP and 0.3 GTP adjusted to pH 7.3 with KOH. The osmolarity of the internal solution was adjusted to 290–305 mOsm, Single cell voltage (current-clamp) was recorded using an dual EPC10 patch-clamp amplifier (Heka). Data was acquired using the Patchmaster software (Heka) and analysed using macros written for the Igor Pro software (Wavemetrics, Wosego, OR).

### Calcium imaging in slices

Experiments were performed in olfactory bulb (OB) slices obtained from 4 to 5 week-old mice. Animals were anesthetized with ketamine/xylazine (0.05–0.15 ml 18 mg/ml, and 2 mg/ml, respectively). OB slices (200 um thick) were prepared as described above (see Slice Preparation). Imaging was carried out at room temperature with a 20X water immersion objective in a submerged recording chamber mounted on the stage of an upright Zeiss Axioskop (Thornwood, NY, USA) equipped with a CCD camera (C2741-08, Hamamatsu Photonics, Hamamatsu, Japan) connected to a frame grabber (LG-3, Scion, Frederick, MD, USA) on a Dell P4 2.4 GHz computer with 1.5 GB RAM with Windows XP Pro. Scion Image software was used for data acquisition and analysis (Scion). Customized macros were written for shutter control (Uniblitz, Vincent Associates, Rochester, NY, USA) and time-lapse imaging. Images were taken every 4 s (396 s total). The recording chamber was continuously perfused with oxygenated Ringer solution and stimuli were applied through a manifold connected to the perfusion system. Stimuli were applied for 40 s. Data is shown as the fractional change in fluorescent light intensity: F/F0 or (F–F0)/F0, where F is the fluorescent light intensity at each point and F0 is the value of emitted fluorescent light before the stimulus application (baseline).

## Supporting Information

Movie S1 (4.15 MB MOV)
